# Comparison of Visit Rates Before vs After Telehealth Expansion Among Patients With Mental Health Diagnoses Treated at Federally Qualified Health Centers

**DOI:** 10.1001/jamanetworkopen.2022.42059

**Published:** 2022-11-15

**Authors:** Megan B. Cole, Eun Kyung Lee, Melissa Davoust, Kathleen Carey, June-Ho Kim

**Affiliations:** 1 Department of Health Law, Policy, and Management, Boston University School of Public Health, Boston, Massachusetts; 2 Ariadne Labs, Brigham and Women’s Hospital and Harvard T. H. Chan School of Public Health, Boston, Massachusetts; 3 Division of General Internal Medicine & Primary Care, Brigham and Women’s Hospital, Boston, Massachusetts

## Abstract

This cohort study assesses visit rates before and after telehealth expansion to assess whether telehealth availability at federally qualified health centers is associated with visit rates for patients with mental health diagnoses.

## Introduction

Telehealth could improve care for patients with low income and mental health needs.^1^ However, despite rapid expansion nationwide,^2^ limited evidence exists on the association between telehealth expansion and access to care for this population. In 2019, only 6% of Massachusetts federally qualified health centers (FQHCs) used telehealth to deliver live, remote mental health services.^3^ By 2020, all Massachusetts FQHCs delivered some mental health services via telehealth, with vast heterogeneity in the extent of adoption.^3,4^ The aim of the study was to assess whether FQHC-level telehealth availability was associated with visit rates for patients with mental health diagnoses enrolled in Medicaid and served by FQHCs.

## Methods

Our cohort study included patients aged 18 to 64 years enrolled in Medicaid with any baseline mental health diagnosis (eg, depression, anxiety, bipolar disorders), who were attributed to Community Care Cooperative (the largest US FQHC-based Medicaid accountable care organization, located in Massachusetts) and had 1 or more FQHC visits within the past 18 months as of each month, including 1 or more visits before telehealth expansion. The Boston University Institutional Review Board deemed the study exempt and waived informed consent because only deidentified data were used. We followed the STROBE reporting guideline.

The primary data source was the 2019 to 2021 Electronic Data Warehouse, which stores electronic health record data from Community Care Cooperative; 11 FQHCs were included and 5 with invalid telehealth data were excluded. The secondary data source was the 2019 American Community Survey. Study outcomes included (1) visit rates among patients with any mental health diagnosis and 1 of the 4 most prevalent mental health diagnoses and (2) having a follow-up visit within 30 days of a mental health–related emergency department visit, which was a contractual accountable care organization quality measure. The treatment group included FQHCs with high availability of telehealth during the COVID-19 pandemic (≥50% of all visits from April 2020 to March 2021; mean [SD], 68.0% [13.9%]), and the comparison group included FQHCs with lower availability of telehealth during COVID-19 (<50% of visits; mean [SD], 25.7% [8.6%]).

The unit of analysis was the patient-month. We used a difference-in-differences approach with negative binomial and linear probability models to examine changes in outcomes for patients at high-telehealth vs low-telehealth FQHCs before (March 2019-February 2020) vs after (April 2020-March 2021) telehealth expansion. Models were adjusted for patient age, sex, clinical risk score, zip code–level digital access, applied study month and FQHC fixed effects, with errors clustered at the FQHC level. Additional details are in eAppendixes 1 to 4 in the Supplement.

A 2-tailed *P* = .05 was considered to be statistically significant. Analyses were performed using Stata, version 17.0 (StataCorp LLC).

## Results

The study included 143 205 person-months among 11 267 patients (55 173 male person-months [38.5%] and 88 032 female person-months [61.5%]) with a mental health diagnosis (Table 1). Visit rates declined across all FQHCs during the COVID-19 pandemic (Table 2). However, high telehealth availability was associated with a larger relative increase in visit rates among patients with mental health diagnoses (incidence rate ratio, 2.07; 95% CI, 1.97-2.17; *P* <.001) compared with lower telehealth availability. Results were similar for patients with specific diagnoses of depression, anxiety, stressor-related, or mood disorders. High telehealth availability was associated with a relative increase of 7.67 percentage points (95% CI, 2.11-13.23; *P* = .007) in the likelihood of having a follow-up visit within 30 days of a mental health–related emergency department visit.

**Table 1.** Baseline Characteristics Among Patients With Mental Health Diagnoses Served at High vs Low Telehealth FQHCs

Characteristic^a^	% Of person-months		
	Total (n = 143 205 person-months)^b^	Served at FQHC with high telehealth availability (n = 100 007 person-months)^b^^,^^c^	Served at FQHC with low telehealth availability (n = 43 198 person-months)^b^^,^^d^
Age, mean (SD), y	37.8 (12.5)	38.1 (12.5)	37.3 (12.6)
Sex			
Female	88 032 (61.5)	64 705 (64.7)	23 327 (54.0)
Male	55 173 (38.5)	35 302 (35.3)	19 871 (46.0)
Race and ethnicity^e^			
Hispanic	48.8	47.9	51.0
Non-Hispanic Black	8.7	7.2	12.7
Non-Hispanic White	37.2	38.9	32.9
Other or multiple races	5.2	6.0	3.3
Primary language^f^			
English	73.1	70.9	77.8
Spanish	22.8	23.6	21.0
Other	4.2	5.5	1.2
Enrolled in Medicaid	100	100	100
Resided in metropolitan urban area^g^	98.8	98.7	98.9
Chronic Illness and Disability Payment System risk score, mean (SD)^h^	2.38 (1.72)	2.31 (1.65)	2.54 (1.87)
Clinical diagnosis			
Diabetes	9.5	10.2	8.0
Chronic obstructive pulmonary disease	6.1	6.7	4.8
Asthma	20.2	19.7	21.1
Hypertension	20.0	20.7	18.5
Hyperlipidemia	14.2	16.6	9.0
Overweight or obese^i^	36.2	37.7	32.8
Morbidly obese	9.5	10.4	7.6
Tobacco use	31.6	31.9	31.1
Alcohol use disorder	12.5	11.7	14.0
Cannabis use disorder	6.4	6.7	5.5
All other substance use^j^	14.6	14.0	15.9
Mental health disorders			
Major depression	49.6	51.9	44.4
Other depression^k^	7.5	8.1	6.1
Anxiety	50.2	50.9	48.4
Attention-deficit/hyperactivity disorder	11.1	12.0	9.0
Autism spectrum disorder	0.7	0.6	0.8
Bipolar disorder	11.8	12.2	10.7
Other mood disorders^l^	16.1	15.2	17.9
Schizophrenia and other psychiatric disorders	5.3	5.9	4.2
Posttraumatic stress disorder, trauma, and stressor-related disorders	38.7	43.6	27.9
Personality disorders	1.0	0.8	1.3
All other mental health disorders^m^	44.3	44.5	43.8
Digital access in household (zip code based)			
Computer, smartphone, tablet, or other device	90.1	90.2	89.9
Internet	84.2	84.3	84.0
Broadband	84.0	84.1	84.0

Abbreviation: FQHC, federally qualified health center.

^a^ Characteristics are shown for the full study population with any mental health diagnosis, although the denominator populations vary by study measure.

^b^ Sample sizes reflect person-months, the unit of analysis. The person-months represent 11 267 unique persons in the full sample, 7756 unique persons in the high telehealth group, and 3411 unique persons in the low telehealth group. Telehealth included real-time video and audio-only visits delivered to remote patients.

^c^ High telehealth group included patients with mental health diagnoses at FQHCs in which 50% or more of all visits to the FQHC in the posttelehealth expansion period were delivered via telehealth (mean [SD], 68.0% [13.9%]).

^d^ Low telehealth group included patients with mental health diagnoses at FQHCs in which less than 50% of all visits to the FQHC in the posttelehealth expansion period were delivered via telehealth (mean [SD], 25.7% [8.6%]).

^e^ Patients self-reported race and ethnicity. Unknown race and ethnicity included 19.7% in the full sample, 16.1% in the high telehealth group, and 27.7% in the low telehealth group. Other races included Asian, American Indian or Alaska Native, Native Hawaiian or other Pacific Islander, or other race.

^f^ Unknown language included 0.97% in the full sample, 0.95% in the high telehealth group, and 1.0% in the low telehealth group. Forty-six languages are included in the other languages group; the most common were Cape Verdean, Portuguese, Arabic, Vietnamese, French, Nepali, and Russian.

^g^ Based on a patient’s zip code, for which metropolitan was defined using the rural-urban commuting area code classification system.

^h^ The Chronic Illness and Disability Payment System is a weighted risk score based on a hierarchal vector of 58 disease categories, which accounts for disease severity within each category. In our study sample, scores ranged from 0.2 to 20.9.

^i^ Overweight or obese is based on having a body mass index (calculated as weight in kilograms divided by height in meters squared) of 25 or greater.

^j^ Other substance use includes opioid use; sedative, hypnotic, or anxiolytic abuse; cocaine use; other stimulant use; hallucinogen use; inhalant use; and other psychoactive substance use.

^k^ Other depression includes diagnoses for other depressive episodes and other recurrent depressive disorders.

^l^ Other mood disorders include dysthymic disorder, other persistent mood (affective) disorders, persistent mood (affective) disorder, and unspecified mood (affective) disorder.

^m^ Other mental health disorders include other psychosis; disruptive, impulse-control, and conduct disorders; obsessive-compulsive and related disorders; history of suicidal ideation, attempts, or self-inflicted injury; dissociative disorders; conversion disorders; somatization disorders; somatoform disorders; and mental disorders not otherwise specified.

**Table 2.** Association Between FQHC-Level Telehealth Availability and Visit Rates Among Patients With Mental Health Diagnoses

	No. of person-months	Marginal effects				Adjusted coefficient for difference-in-differences (95% CI)^a^^,^^b^	*P* value
		High-telehealth FQHC^c^		Low-telehealth FQHC^d^			
		Pretelehealth expansion visits^e^	Posttelehealth expansion visits^f^	Pretelehealth expansion visits^e^	Posttelehealth expansion visits^f^		
Follow-up visit within 30 d of mental health emergency department visit, No./total No. (%)^g^	5207	2097/2591 (80.9)	667/810 (82.3)	1052/1324 (79.5)	339/482 (70.3)	7.67 (2.11-13.23)	.007
No. of visits per 100 patients per mo among patients with mental health diagnosis^h^							
Any mental health diagnosis	143 205	88.8	51.3	138.1	37.2	2.07 (1.97-2.17)	<.001
Depression diagnosis	71 665	105.7	65.4	157.2	43.7	2.30 (2.15-2.46)	<.001
Anxiety diagnosis	69 723	102.2	62.6	161.3	43.8	2.19 (2.06-2.34)	<.001
Posttraumatic stress disorder, stressor, and trauma disorders diagnosis	55 129	106.9	68.1	276.8	76.7	2.23 (2.06-2.42)	<.001
Bipolar and other mood disorders diagnosis	32 574	109.9	75.9	232.4	70.9	1.83 (1.68-1.99)	<.001

Abbreviation: FQHC, federally qualified health center.

^a^ The difference-in-differences coefficient for the quality of care follow-up measure is from a linear probability model, where the coefficient is reported on an absolute percentage point scale; a value greater than 0 indicates that telehealth was associated with an increase in the measure. The difference-in-differences coefficients for number of visits are from negative binomial models, where coefficients are reported as a relative incidence rate ratio; a value greater than 1 indicates that telehealth was associated with an increase in the measure.

^c^ High telehealth group included patients with mental health diagnoses at FQHCs in which 50% or more of all visits to the FQHC in the posttelehealth expansion period were delivered via telehealth (mean [SD], 68.0% [13.9%]).

^d^ Low telehealth group included patients with mental health diagnoses at FQHCs in which less than 50% of all visits to the FQHC in the posttelehealth expansion period were delivered via telehealth (mean [SD], 25.7% [8.6%]).

^e^ Pretelehealth expansion period is March 2019 to February 2020.

^f^ Posttelehealth expansion period is April 2020 to March 2021.

^g^ The quality of care follow-up measure excludes the first 6 months of the COVID-19 pandemic due to the rolling, look-back nature of Healthcare Effectiveness Data and Information Set measure calculation. Denominators shown are larger in the pretelehealth vs posttelehealth expansion period due to having more observation time, and thus more person-months, in the pretelehealth expansion period.

^h^ Number of visit measures excludes March 2020. Number of visits for any mental health diagnosis includes any FQHC visit among patients with any of the mental health diagnoses listed in Table 1. Visits by subdiagnosis represent any FQHC visit among patients with those specific diagnoses (diagnoses are not mutually exclusive or exhaustive).

## Discussion

High telehealth availability at FQHCs was associated with better care engagement during the COVID-19 pandemic for patients enrolled in Medicaid who had mental health diagnoses despite declines in overall visit rates across all FQHCs. Study limitations included having only 1 year of posttelehealth expansion data, potential data reporting error, unmeasured confounding due to simultaneous implementation of telehealth and other pandemic-related programs, and potential limited generalizability outside Massachusetts. As supported by other research,^5,6^ this study suggests that care delivery models that support telehealth as part of mental health care may be associated with improved engagement for patients enrolled in Medicaid.

## Notes

Supplement. **eAppendix 1.** FQHCs in Study Sample Compared to FQHCs Excluded From the Study Sample **eAppendix 2.** Defining Telehealth **eAppendix 3.** Defining High Versus Low Telehealth FQHCs **eAppendix 4.** Identifying Video vs Audio-Only Telehealth Visits
