# Supplementary material for: Comparison of Visit Rates Before vs After Telehealth Expansion Among Patients With Mental Health Diagnoses Treated at Federally Qualified Health Centers
Source: JAMA Netw Open. 2022 Nov 15;5(11):e2242059. doi: 10.1001/jamanetworkopen.2022.42059 (PMC9667322; doi:10.1001/jamanetworkopen.2022.42059)
Supplement: Supplement. — eAppendix 1. FQHCs in Study Sample Compared to FQHCs Excluded From the Study Sample eAppendix 2. Defining Telehealth eAppendix 3. Defining High Versus Low Telehealth FQHCs eAppendix 4. Identifying Video vs Audio-Only Telehealth Visits [file jamanetwopen-e2242059-s001.pdf]

Supplementary Online Content

Cole MB, Lee EK, Davoust M, Carey K, Kim JH. Comparison of visit rates before vs after telehealth expansion among patients with mental health diagnoses treated at federally qualified health centers. *JAMA Netw Open.* 5(11):e2242059. doi:10.1001/jamanetworkopen.2022.42059

eAppendix 1. FQHCs in Study Sample Compared to FQHCs Excluded From the Study Sample
eAppendix 2. Defining Telehealth
eAppendix 3. Defining High Versus Low Telehealth FQHCs
eAppendix 4. Identifying Video vs Audio-Only Telehealth Visits

This supplementary material has been provided by the authors to give readers additional information about their work.

© 2022 Cole MB et al. *JAMA Network Open.*

eAppendix 1. FQHCs in study sample compared to FQHCs excluded from the study sample

Table A1-1. Comparison of patient characteristics at C3 FQHCs that were included in the study sample vs C3 FQHCs that were excluded due to invalid telehealth data

	Included	Excluded
Characteristic	(N=143,205 person-months)	(N=48,213 person-months)
Age, mean (SD)	37.80	38.14
Female, %	61.30%	60.97%
Race/ethnicity, %*		
White, non-Hispanic	37.20%	37.43%
Non-White	62.80%	62.57%
Primary language is English, %[b]	73.10%	73.99%
CDPS risk score, mean (SD)	2.38	2.41
Clinical diagnoses, %		
Diabetes	9.50%	9.38%
COPD	6.10%	5.17%
Asthma	20.20%	15.94%
Hypertension	20.00%	25.27%
Hyperlipidemia	14.20%	15.62%
Overweight or obese	36.20%	35.39%
Morbidity obese	9.50%	5.57%
Tobacco use	31.60%	31.82%
Alcohol use disorder	12.50%	13.46%
Cannabis use disorder	6.40%	4.76%
All other substance use	14.60%	19.53%
Mental health disorders		
Major depression	49.60%	51.27%
Other depression	7.50%	9.70%
Anxiety	50.20%	51.76%
ADHD	11.10%	7.66%
Autism Spectrum Disorder	0.70%	0.81%
Bipolar disorder	11.80%	10.73%
Other mood disorders	16.10%	19.58%
Schizophrenia and other psychiatric disorders	5.30%	6.51%
PTSD, trauma, and stressor-related disorders	38.70%	34.43%
Personality disorders	1.00%	0.97%
Digital access in household (zip code-based), %		
Computer, smart phone, tablet, or other device	90.10%	90.20%
Internet	84.20%	84.55%
Broadband	84.00%	84.33%

© 2022 Cole MB et al. *JAMA Network Open.*

Table A1-2. Comparison of FQHCs characteristics: study sample vs Massachusetts vs US FQHCs (2020)

	FQHCs in study sample (N=11 FQHCs)	FQHCs in Massachusetts (N=37 FQHCs)	FQHCs in US (N=1375 FQHCs)
Age group, %			
Children (<18)	17.2%	16.5%	22.2%
Adults (18-64)	69.5%	70.0%	64.1%
Elderly (>=65)	13.0%	12.2%	11.3%
Female, %	56.0%	54.4%	56.5%
Non-white, %	60.4%	65.4%	59.0%
Primary language other than English, %	31.6%	33.1%	20.9%
Enrolled in Medicaid, %	42.9%	43.6%	41.9%
Under 100% of federal poverty level, %	53.0%	62.9%	64.5%
Urban service area, %	81.8%	81.1%	58.0%
Unique patients served/FQHC (annual mean)	24,255	20,558	20,793
Using any real time telehealth in 2020, %	100.0%	100.0%	97.0%
EHR at all sites, %	100.0%	100.0%	99.6%

Note: Characteristics represent all FQHC patients, as reported in the 2020 Uniform Data System (UDS). Characteristics are not limited to patients covered by Medicaid. In contrast, our study population includes only Medicaid enrollees age 18-64. Nonetheless, these data compare characteristics at the FQHC facilities based on available data. These data may inform generalizability of our study results – the association between telehealth availability and service use may or may not differ across FQHCs with different characteristics.

© 2022 Cole MB et al. *JAMA Network Open.*

eAppendix 2. Defining telehealth

For encounters that were otherwise flagged as patient visits within the EHR, we classified a visit as a telehealth visit if any of the following were true:

Table A2-1. Codes used to identify telehealth visits

Encounter type or encounter type description contained the following text string	CPT codes contained in charge fields included the following
Video*<br>Zoom*<br>Teleh*<br>Telemed*<br>Phon*<br>Virtual*<br>Tele Ofc*	99441-99443 (with modifier GT, 95, or V3)<br>99211-99215; 99201-99205

Further, we excluded from the telehealth definition instances where encounter type included: “COVID” or “triage.”

All encounter types, encounter type descriptions, and charge codes were reviewed by two research team members, our analytic vendor, and by the C3 ACO leadership team. This included manually reviewing and classifying over 5,500 encounter type descriptions.

© 2022 Cole MB et al. *JAMA Network Open.*

eAppendix 3. Defining high versus low telehealth FQHCs

To categorize FQHCs are “high” versus “low” telehealth FQHCs, for each FQHC, we examined the percentage of all FQHC visits that were delivered via telehealth during our post-period (i.e. April 2020 – March 2021). For qualitative validation, we compared these estimates to two external sources of FQHC-level telehealth availability, which weren’t specific to mental health: (1) biweekly survey data, reported by every FQHC to HRSA during the COVID-19 pandemic, that reported the percentage of all medical visits delivered via telehealth and (2) a C3 ACO-administrated telehealth assessment, that qualitatively categorized each FQHC’s telehealth adoption level. In instances where our study population estimates did not qualitatively align with the other data sources (i.e., in instances where EHR data likely underreported telehealth use, as rates were very low), we considered these EHR classifications invalid and excluded those FQHCs from our sample.

Among FQHCs with valid EHR telehealth data, we examined the distribution of “percentage telehealth visits among all visits” from April 2020 – March 2021. Percentages ranged from 11% to 88%. The 50% threshold was where the two groups naturally diverged: within the study population, all FQHCs had either >53% or <34% of visits delivered via telehealth (i.e. no FQHCs delivered 34-53% of visits via telehealth – it was always higher or lower than this range). In the “high telehealth” group, the percentage ranged from 54-88% of visits (mean=68.0%). In the “low telehealth” group, the percentage ranged from 11-34% of visits (mean=25.7%).

© 2022 Cole MB et al. *JAMA Network Open.*

eAppendix 4. Identifying video vs audio-only telehealth visits

Identifying whether telehealth visits are audio only versus video is challenging, as data are often missing in EHRs and the validity of these EHR and claims measures is not well understood. This has recently been described in the literature (Hailu et al, 2022).

Nevertheless, we were able to estimate the distribution of telehealth visits that were audio only vs telehealth. We did this using "encounter type" codes in the EHRs and charge data from FQHC billing systems. For all patient encounters that were otherwise flagged as telehealth, we further classified telehealth visits as "video" vs "audio-only" if any of the following were true:

Table A4-1. Codes used to differentiate video vs audio telehealth visits

	Encounter type or encounter type description contained the following text string	CPT codes contained in charge fields include the following
Video visits	video (e.g., "Video – Medical Short 20" ; "Psych Video 20 F/U Visit")<br><br>zoom (e.g., "Zoom visit")<br><br>virtual (e.g., "Virtual New Patient Initial" ; "Virtual Behavioral Health Visit")	99211-99215; 99201-99205
Audio-only visits	phon* (e.g., "Phone Preventive Care 30" ; "Phone Visit 20" ; "Psych Phone 20 F/U Visit")	99441-99443

Within our study population, of all FQHC encounters (i.e. visits) that were otherwise flagged as telehealth, 55% were classified as either video or audio-only. The remaining 45% of telehealth visits could not be classified. Of the 55% that were classified, 36% were classified as video and 64% were classified as audio-only. We report these statistics within our manuscript, but do so with the caveat that these classifications are subject to numerous limitations.

All encounter types, encounter type descriptions, and charge codes were reviewed by two research team members, our analytic vendor, and by the C3 ACO leadership team.

© 2022 Cole MB et al. *JAMA Network Open.*
